# Conserved Thalamic Astrocytic Pathology in Mice and Patients Links TBI to Epilepsy

**DOI:** 10.21203/rs.3.rs-11243479/v1

**Published:** 2026-10-23

**Authors:** Federico Moro, Ilaria Lisi, Edward Needham, Elaine O'Loughlin, Rossella Di Sapia, Karthik Chary, Stefano Fabrizio Columbro, Anastasia Lubarsky, Luther Loose, Edoardo Mazzone, Sam Husarik, Karinn Sytsma, Firas Kobeissy, Edoardo Micotti, Annamaria Vezzani, Virginia F.J. Newcombe, Emmanuel A. Stamatakis, Valentina Bonetto, Teresa Ravizza, Laura Lubbers, Kevin K. W. Wang, William E. Haskins, Amber L. Nolan, David K. Menon, Elisa R. Zanier

## Abstract

Post-traumatic epilepsy (PTE) can emerge months to years after traumatic brain injury (TBI) and is a major cause of morbidity, yet the pathological processes that distinguish TBI progressing to epilepsy remain poorly defined. Here, we combined longitudinal multimodal imaging, electrophysiology, neuropathology, and human imaging and postmortem analyses to identify cross-species features associated with PTE. In a mouse model of PTE-prone TBI, injury produced widespread brain pathology, whereas animals developing PTE showed disproportionate thalamic atrophy, white-matter abnormalities with reduced optic tract fractional anisotropy, and persistent metabolic alterations. Histological analyses revealed pronounced thalamic astrocytic reactivity, reduced cytoskeletal complexity, and impaired expression of proteins involved in glutamate homeostasis. Using an early ECoG signature to prospectively stratify PTE risk, we found that astrocytic simplification and accumulation of insoluble GFAP were already present during the latent period before spontaneous seizure onset. In patients with PTE, thalamic volume loss and reduced posterior thalamic radiation fractional anisotropy independently improved PTE classification beyond clinical variables. PTE patients also showed reduced pulvinar-centered thalamocortical functional connectivity involving visual and default mode networks. Postmortem tissue further demonstrated pronounced astrocytic reactivity and reduced astrocytic complexity in cortex and thalamus. In contrast, IBA1-defined microglial reactivity did not distinguish PTE from non-PTE at the chronic time points examined. Together, these findings identify a conserved thalamocortical phenotype associated with PTE and show that astrocytic remodeling emerges before spontaneous seizures. The convergence of cellular pathology with structural, metabolic, and functional abnormalities identifies the thalamus as a candidate hub of post-traumatic epileptogenesis and provides translational markers for tracking disease progression after TBI.

